# Application of Pomegranate by-Products in Muscle Foods: Oxidative Indices, Colour Stability, Shelf Life and Health Benefits

**DOI:** 10.3390/molecules26020467

**Published:** 2021-01-17

**Authors:** Arun K. Das, Pramod Kumar Nanda, Nilabja Roy Chowdhury, Premanshu Dandapat, Mohammed Gagaoua, Pranav Chauhan, Mirian Pateiro, Jose M. Lorenzo

**Affiliations:** 1 Eastern Regional Station, ICAR-Indian Veterinary Research Institute, Kolkata 700037, India; arun.das@icar.gov.in (A.K.D.); pdandapat@gmail.com (P.D.); 2 Department of Veterinary Biochemistry, West Bengal University of Animal and Fishery Sciences, Kolkata 700037, India; drnrc1996@gmail.com; 3 Food Quality and Sensory Science Department, Teagasc Food Research Centre, Ashtown, Dublin 15 D15 DY05, Ireland; gmber2001@yahoo.fr; 4 Division of Livestock Products Technology, ICAR-Indian Veterinary Research Institute, Izatnagar, Bareilly 243122, India; dr.pranav.chauhan@gmail.com; 5 Centro Tecnologico de la Carne de Galicia, Rua Galicia N° 4, Parque Tecnologico de Galicia, San Cibrao das Vinas, 32900 Ourense, Spain; mirianpateiro@ceteca.net; 6 Area de Tecnologia de los Alimentos, Facultad de Ciencias de Ourense, Universidad de Vigo, 32004 Ourense, Spain

**Keywords:** pomegranate, bioactive compounds, lipid and protein oxidation, meat, fish, shelf life

## Abstract

In recent years, considerable importance is given to the use of agrifood wastes as they contain several groups of substances that are useful for development of functional foods. As muscle foods are prone to lipid and protein oxidation and perishable in nature, the industry is in constant search of synthetic free additives that help in retarding the oxidation process, leading to the development of healthier and shelf stable products. The by-products or residues of pomegranate fruit (seeds, pomace, and peel) are reported to contain bioactive compounds, including phenolic and polyphenolic compounds, dietary fibre, complex polysaccharides, minerals, vitamins, etc. Such compounds extracted from the by-products of pomegranate can be used as functional ingredients or food additives to harness the antioxidant, antimicrobial potential, or as substitutes for fat, and protein in various muscle food products. Besides, these natural additives are reported to improve the quality, safety, and extend the shelf life of different types of food products, including meat and fish. Although studies on application of pomegranate by-products on various foods are available, their effect on the physicochemical, oxidative changes, microbial, colour stabilizing, sensory acceptability, and shelf life of muscle foods are not comprehensively discussed previously. In this review, we vividly discuss these issues, and highlight the benefits of pomegranate by-products and their phenolic composition on human health.

## 1. Introduction

Muscle foods, in particular meat and fish, are considered as excellent sources of high quality proteins containing balanced amino acids, vitamins (B group), minerals, and a number of other nutrients [1,2,3,4]. Even though having high nutrient contents, muscle foods also contain metal catalysts, haem pigment, various oxidizing agents, and abundant unsaturated fatty acids, which are unstable, especially when exposed to extreme environmental conditions such as constant high temperature, air, and light [5]. These food products with high water content and moderate pH are perishable in nature, hence cannot be stored for longer periods without any preservatives [6]. The susceptibility of these products to spoilage results from microbial activities and undesirable chemical changes, such as oxidation of muscle proteins and lipids during storage [7,8,9]. Lipid oxidation of muscle foods results in extensive colour loss, structural damage to protein, and production of rancid or unpleasant flavours [4,10,11]. These changes negatively affect the sensory quality, nutritional value, and consumer acceptability, and consequently shorten the shelf life of muscle foods [12,13,14,15,16,17]. As far as protein oxidation in muscle foods is concerned, it changes the amino acid structures, leading to the formation of carbonyl and reduction of sulfhydryl content [18]. Oxidative changes ultimately affect the tenderness and water holding capacity of muscle food during storage [19,20]. 

There are different ways to prevent the microbial activities and oxidative deterioration of muscle foods. Synthetic chemicals are mostly used to inhibit such types of changes and minimize the formation of toxic compounds such as cholesterol oxidation products [21,22]. However, in recent times, natural preservatives extracted from various agrifood wastes are being explored by food processors that not only contain antimicrobials and antioxidants but also are abundant, cheap and environment friendly. In addition, many plant parts (fruits, roots, bark, leaves) and their coproducts are also reported to provide a rich source of natural bioactive compounds (polyphenolic, dietary fibre and flavonoids) that not only play a vital role in inhibiting oxidative changes (antioxidants) but also help in suppressing microbial growth (antimicrobial), thereby preventing several diseases [23,24]. Again, consumers worldwide also prefer these natural preservatives, which are considered as safe and exert positive health effects over synthetic chemicals that have toxicity and health risks [5,25,26]. 

Pomegranate (*Punica granatum* L.), a member of the family Punicaceae, is a deciduous shrub or small tree widely cultivated in the Middle East, European, and Southeast Asian countries [5]. Each and every part of pomegranate plant (leaves, stem, fruits, bark and roots) possess numerous bioactive compounds like phenolic compounds, including hydrolysable tannins (pedunculagin, punicalin, punicalagin, and ellagic and gallic acids), flavonoids (catechins, anthocyanins, and other complex flavonoids) and complex polysaccharides [27,28]. It is a fruit, commonly known as ‘‘seeded apple’’ or ‘‘granular apple’’, highly valued and consumed worldwide for its pleasant taste, nutritional values, and medicinal properties [29]. Pomegranate fruit is used in fruit processing and beverage industry for preparation of juice and soft drinks, and during the production process, a large quantity of fruit-derived low-cost nonedible waste (mostly peel and seed) is generated. These wastes are valuable sources of bioactive compounds and could either be used as functional food ingredients or as food additives, nutraceuticals, and supplements to enrich phenolic content in diets [29,30]. These bioactive compounds, apart from being natural, exert antioxidant and antimicrobial activity and are reported to improve the quality, safety, and extend the shelf life of different types of food products such as oils [5], meat [23,31,32,33], fish [34,35,36], and dairy products like cheese, curd, fermented milk [37], cereal based cookies [38]. Studies on application of pomegranate by-products on various muscle foods are available, but its effect on the physicochemical, microbial, colour stabilizing, oxidative changes, sensory acceptability, and shelf life has not previously been comprehensively discussed. In this review, the authors vividly discuss these issues, and highlight the use of pomegranate by-products and the effects of their phenolic composition on human health. 

## 2. Pomegranate Fruit and Its By-Products

Pomegranate fruit, regarded as superfruit of the next generation, is quite popular throughout the globe due to its sweetness, acidic juices, and extensive medicinal properties, including antimicrobial, antioxidant, antimutagenic, antihypertensive and hepatoprotectant properties [37,39,40]. The outer hard covering of the fruit is red-purple in colour and called pericarp, whereas the inner spongy wall is called mesocarp (white “albedo”), where seeds are attached. A mature pomegranate fruit measures about 6–10 cm in diameter, weighs 200 g on an average, and usually contains 50% peel, 40% arils and 10% seeds. Further, the pomegranate fruit processing establishments also generate a large quantity of by-products/wastes after extraction of juice from the fruits. The wastes are called pomace or bagasse, which is nothing but a mixture of peel, seed, and mesocarp, which remains underutilised in food industry. These wastes could be fortified in various food systems for design and development of healthy functional foods with improved quality and shelf life, offering health benefits [41,42,43]. The fruit is a good source of dietary fibre (both soluble and insoluble), but contains no cholesterol or saturated fats and is low in sugar. In addition, it also contains about 80–85 calories per 100 g, vital minerals (potassium, copper, manganese, and zinc), and vitamin C and B complex groups, such as pantothenic acid (vitamin B-5), folates, pyridoxine, and vitamin K [44]. The proximate composition, major micronutrients, vitamins, and polyphenolic contents of pomegranate peel and seed powder are represented in Table 1.

## 3. Polyphenolic and Flavonoid Compounds in Pomegranate Fruit By-Products

**Figure 1:**
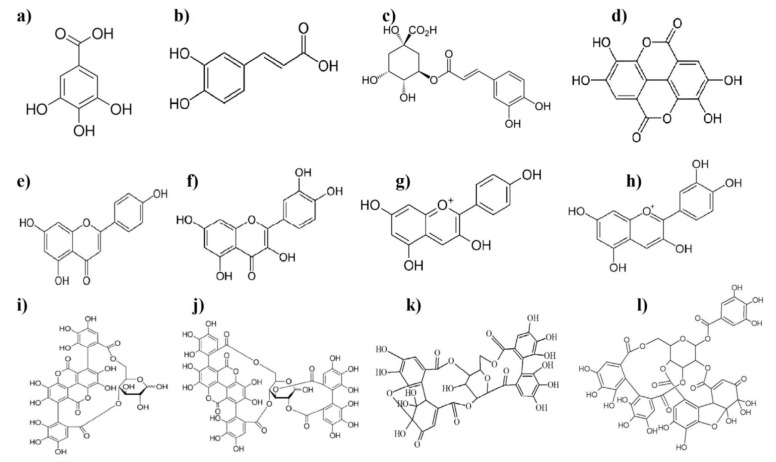
Chemical structure of major phenolic compounds from pomegranate fruit: (**a**) gallic acid, (**b**) caffeic acid, (**c**) chlorogenic acid, (**d**) ellagic acid, (**e**) apigenin, (**f**) quercetin, (**g**) pelargonidin, (**h**) cyanidin, (**i**) punicalin, (**j**) punicalagin, (**k**) granatin A, and (**l**) granatin B.

Polyphenols are a structural class of organic chemicals containing large multiples of phenolic structural units. The chemical structure of major phenolic compounds from pomegranate fruit and its by-products are presented in Figure 1, including ellagitannins (ellagic acid, punicalagin, gallic acid, punicalin), anthocyanins (cyanidin and pelargonidin), phenolic acids like caffeic acid, chlorogenic acid, and flavonoids (quercetin).

The phenolics and polyphenolic compounds in pomegranate fruit and its by-products (seed, juice, pomace, and peel) have been studied in detail by several workers. Not only the succulent testa of pomegranate fruit, but the peels are also sources of phenolics, pectin, and complex polysaccharides, whereas the arils are rich in water, sugars, organic acids, and polyphenolic compounds, especially flavonoids. In addition, the major phenolic compound in the pomegranate juice is anthocyanin, the pericarp, and mesocarp are sources of hydrolysable tannins [37,47]. The by-products also contain important compounds and chemicals that are valuable sources of antioxidants, tannins, dynes, and alkaloids [29,48]. The major chemical constituents, phenolics, and organic compounds reported in different pomegranate plant parts are presented in Table 2.

## 4. Antioxidant Activity of Pomegranate Wastes at Cellular Levels

Reactive oxygen species (ROS) is actually beneficial for the health as it contributes to the immunity of an individual via respiratory bursts, etc. [49]. These ROS or the free radicals deteriorate the integrity of proteins, lipids, and genetic materials (DNA and RNA) [50], as a result of which several degenerative diseases, cardiovascular diseases, and metabolic diseases like diabetes, cancer, and severe immune suppression of the body, may occur [51,52]. Edible and nonedible parts of the pomegranate fruit are reported to have strong antioxidant effects, which are helpful to neutralise the free radicals, i.e., the ROS, and protect the cells and the tissues, ultimately preventing ageing and reducing the chances of noncommunicable disease occurrences [53,54]. The ellagitannins, derived from the pomegranate fruit, have a cryoprotective effect on the neuronal cells (Neuro-2a) challenged with hydrogen peroxide [55]. In fact, the ellagitannins get converted into urolithin A in the human gut [56], which increases peroxiredoxin 1 and 3 expressions, enhancing the cryoprotective properties. Additionally, dose-dependent inhibition of oxidising enzymes like monoamine oxidase A and tyrosinase are also experienced [55].

## 5. Antimicrobial Activity

### 5.1. Antibacterial Activity

The polyphenolic compounds (flavonoids, tannins) obtained from the extracts of pomegranate peel and other wastes are a good source of antibacterial components that help in combating the bacterial growth responsible for foodborne diseases and food spoilage [57,58]. These polyphenols form complexes with the bacterial cell wall proteins [59] and interact with the sulfhydryl groups of the extracellular protein matrix of the bacteria to inhibit their activities and ultimately lyse them [57]. The antibacterial efficacy of various pomegranate by-products (peels, pulp and other wastes) are demonstrated via in vitro studies using bacterial cultures with the help of agar gel diffusion assays or minimum inhibitory concentration assays [41]. 

Similarly, tannins derived from the pomegranate extracts not only disrupt the cell–microbial adhesion, but also are reported to hinder the mineral consumption by the bacteria [60]. Even the biofilm production and mobility of *Escherichia coli* (especially uropathogenic and enterohaemorrhagic strains) are greatly reduced by the pomegranate peel extracts [61]. Abdollahzadeh et al. [62] found higher degrees of inhibitory effects of methanolic extracts of pomegranate peel against *Staphylococcus aureus*, *Staphylococcus epidermidis*, *Lactobacillus acidophilus*, *Actinomyces viscosus*, *Streptococcus sanguinis*, etc., suggesting their use as an antibacterial agent in controlling oral infections. Likewise, clinical isolates of methicillin resistant and susceptible *S. aureus* strains are also reported to be highly susceptible to CuSO_4_ enriched and combined with pomegranate peels [63]. 

Pomegranate pulp extracts (PPE) are also remarkably effective against Gram-positive (*Listeria monocytogenes* and *Listeria innocua*) and Gram-negative (*Pseudomonas aeruginosa* and *Salmonella* sp.) bacteria [64]. In addition, these extracts are also effective against various oral infections caused by bacteria like *S. sanguinis* and *Streptococcus mitis* [65]. Thus, PPE can not only act as a low-cost phytomedicine for human health development, but it can also reduce the usage pattern and associated risk factors involved with antibiotic consumption [66].

### 5.2. Antifungal Activity

The most common causes of food spoilage are caused by mycotoxins secreted by fungi, causing economic losses and health threats to individuals. In several studies, pomegranate peels and seeds extracts have shown promising antifungal properties and can be used as antifungals, replacing their synthetic alternatives [67]. The presence of antifungal compounds, especially with high concentrations of punicalagin, in hydroalcoholic crude extracts of pomegranate wastes have demonstrated potent antifungal activity against *Trichophyton mentagrophytes*, *Trichophyton rubrum*, *Microsporum canis*, and *Microsporum gypseum* [68]. Strong antifungal activity of pomegranate PPE has also been recorded in in vitro studies against *Botrytis cinerea*, *Penicillium digitatum*, and *Penicillium. expansum* [69]. Likewise, methanolic extract of pomegranate peel showed activity against *Candida albicans* [62] and *Aspergillus niger*, *Candida utilis*, *Saccharomyces cerevisae* [70]. Furthermore, pomegranate extract gels are also reported as effective against oral infections of fungal origin (like *Candida* spp.) [65]. 

## 6. Health Benefits

**Figure 2:**
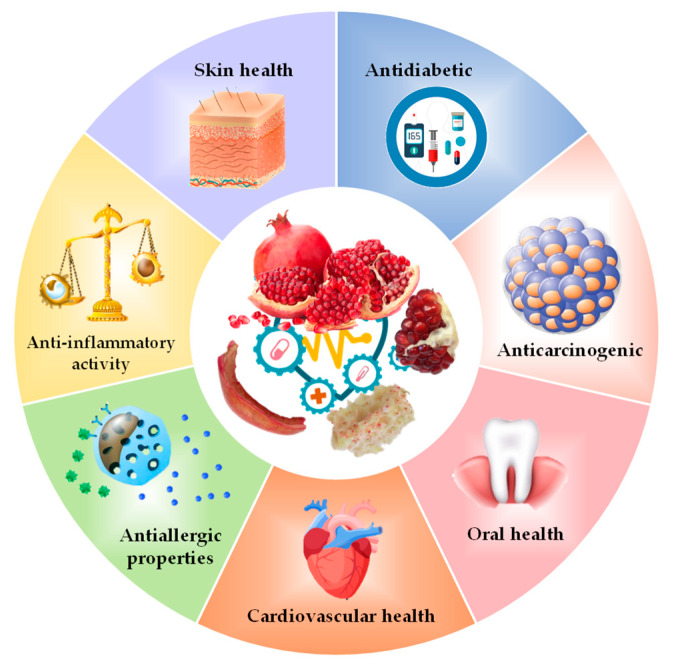
Health benefits associated with pomegranate by-products.

Pomegranate has been a very useful natural source for building health (Figure 2). The consumption of its juice, obtained from arils, improves the antioxidant status in humans by increasing the glutathione levels (22.6%) in erythrocytes and decreasing lipid peroxidation (24.4% malondialdehyde) and protein oxidation (19.6% carbonyl) [71]. The bioactive compounds (phenolic compounds and organic acids) found in the testa, pericarp (peel), seeds, etc., not only act as antioxidative, antitumoral, and antihepatotoxic agents, but also have medicinal, nutritional, and pharmaceutical properties as well. These compounds have positive effects on cardiovascular, neuronal, renal, and immune systems, which in turn helps in preventing various diseases, thus offering health benefits [46].

Further, the components from pomegranate peel and seeds, due to their anticancer properties, are capable of fighting a variety of chronic diseases such as colon and prostate cancer, melanogenesis (skin cancer), breast cancer, and stomach ulcers [23,72]. A summary of different components of pomegranate fruits on various health issues is presented in Table 3.

### 6.1. Gut Modulation and Prebiotic Effect

The consumption of pomegranate fruit helps in modulating the gut microbiota [37]. The most abundant polyphenolic compound, ellagitannins, are metabolised in the gut to form ellagic acid, which in turn is converted into urolithin A and urolithin B by the colon microbiota [56]. These two compounds act as prebiotic and are responsible for the growth of bacterial bloom of *Lactobacillus* sp. and *Bifidobacterium* sp. in the gut [73], which in turn prevents the growth of *Bacteroides fragilis* and *Clostridium* sp., the harmful bacteria of *Enterobacteriaceae* [73]. Likewise, the gallic acid and ellagic acid are utilised by the faecal bacteria to produce urolithin, helpful for producing short-chain fatty acids (acetate, propionate, and butyrate) [74]. Punicalagins help in inhibiting growth of pathogenic *Clostridia* and *S. aureus* in vitro [75]. Pomegranate juice fortified with the pericarp extract and lactic acid bacteria also show promising results in terms of higher survival rate of the gut-acting bacteria and higher bioaccessibility of phenolic compounds due to the metabolism of ellagitannins, epicatechin, and catechin metabolism in the gut lumen [76].

**Table 3:** Effects of pomegranate fruits and its extracts on human health.

Pomegranate Fruit	Health Complication/Patient Type	Effect after Pomegranate Supplementation		Ref.
Whole fruit extract	Obesity	Lesser Inflammation Body weight Blood glucose Total cholesterol Low-density lipoprotein (LDL)	Higher High-density lipoprotein (HDL)	[77]
Juice	Patient undergoing haemodialysis	Lesser Blood pressure Serum Triglycerides Oxidative stress Inflammation	Higher HDL	[42]
Juice	Type 2 diabetes	Lowered Serum erythropoietin		[78]
Microencapsulation	Women with acute coronary syndrome	Reversal of high-density lipoprotein- induced endothelial dysfunction Lowered postprandial triglyceridemia		[79]
Pericarp extract	Type 2 diabetes	Lesser Free fatty acids Blood glucose	Higher Antioxidative potential	[80]
Pericarp extract	Dyslipidaemia	Lesser Systolic blood pressure LDL Total cholesterol	Higher HDL	[81]
Juice	Active adults	Visual memory skills maintenance		[82]
Juice	Type 2 diabetes	Lesser blood pressure		[83]
Juice	Active healthy men	Lesser systolic pressure Lesser creatinine		[84]
Seed oil	Type 2 diabetes	Lesser blood sugar		[85,86]
Juice	Endurance-based athletes	Lesser body fat and protein degeneration		[87]

### 6.2. Antidiabetic and Antiosteoporotic Activity

Primarily, it has been seen that there exists a relation between extracts from pomegranate juice or its by-products and type 2 diabetes [88]. Studies have also depicted the association of pomegranate peel with the type 2 diabetes markers [48,89,90]. The antidiabetic activity of pomegranate rind and aril extracts have also been studied. It has been found that the pomegranate juice/extracts reduce the lipid peroxidation and oxidative stress either by directly scavenging the free radicals or enhancing the α-glucosidase inhibitory activity to manage type 2 diabetes and associated complications [48,88,91].

Physiologically, pomegranate-derived polyphenolic compounds lower blood glucose level, increase glycogen synthesis in liver, elevate insulin secretion, and enhances glucose tolerance [48]. In fact, α-amylase is an enzyme that is initially required for hydrolysis of carbohydrate foods, particularly starch, into smaller molecules before they degrade into glucose molecules. Pomegranate, mostly its rind and aril extracts, inhibit the enzymatic activity of α-amylase, which in turn delays the digestion of carbohydrate foods and reduces the glucose release in blood, responsible for the postprandial serum glucose levels in human body [92]. The anti-α-amylase property of pomegranate also varies depending upon the extraction process. It has been reported that acetone extract of pomegranate peel has 3.5 times more anti-α-amylase property, which may have more potent antidiabetic property than those displayed by the hydroalcoholic aqueous extracts [90]. In addition to these effects, pomegranate extracts also induce osteoblast differentiation in bone tissues [93,94]. Punicalagin, as seen in in vitro studies, blocks osteoclast differentiation [95]. Therefore, regular consumption of pomegranate is helpful for bone health, reducing the chances of osteoporosis occurrence [94].

### 6.3. Pomegranate Role in Dermatology

Being a good source of natural antioxidants, phenolic compounds from pomegranate extracts are considered useful for skin care due to its protective effects against ROS, so may be used in case of keratinocytes [96]. Pomegranate oil and extracts also show photochemo-preventive effects by blocking solar ultraviolet (UV) radiation, particularly its UVB (290–320 nm) component-mediated DNA and protein damage, increasing tropoelastin levels and degrading the extracellular matrix proteins in skin tissue [97]. Previously published work demonstrates that pomegranate peel extract, although exhibiting no growth-supporting effect on keratinocytes, simulates type I procollagen synthesis, which in turn helps in promoting skin (dermis and epidermis) repair [98]. In another in vitro and animal study, the protective effect of pomegranate extract and juice, applied either topically or through oral consumption, against UVB induced skin damage (erythema) and alteration of the composition of skin microbiota in healthy women has also been reported [99]. 

### 6.4. Anticarcinogenic Activity

Various in vitro and in vivo studies suggest the anticarcinogenic activity of pomegranate fruit and its extracts. The mode of action against cancer growth and promotion is by modulating multiple signalling pathways. For example, the extracts block NF-κB activity in vitro in the case of prostate cancer tissues [100] and renal cell carcinoma [101]. Punicalagin, derived from pomegranate extracts, exhibits antiproliferative activity by inducing apoptosis of the cancer cells [102]. Via this mechanism, this phenolic compound acts against papillary thyroid carcinoma cells [103], proliferation of lung carcinoma cell lines, and breast and cervical cancer cell lines [104]. The pomegranate extracts target cancer tissues by targeting or blocking the functions of certain molecules engaged in intercellular and extracellular matrix adhesions, proinflammatory and proangiogenic molecules, modulating cytoskeleton of the tumour cells and chemotactic compounds at cellular level [105].

### 6.5. Role in Cardiovascular Problems

A recently published article suggest that pomegranate rich diet may help a person to avoid cardiovascular diseases [40]. In fact, the polyphenolic compounds derived from pomegranate are able to decrease serum cholesterol and intima-media thickness, reduce lipid peroxidation levels and blood pressure, and decrease nitric oxide concentrations [106,107]. Further, in a recent study, pomegranate has also been reported to reduce angiotensin-converting enzyme activity (possibly helping to stay immune against COVID-19, as the virus also uses ACE2 receptors) [108]. Punicalagin can active Forkhead box O1 (Fox O1), which helps to prevent vascular dysfunction and elevates cellular Paraoxonase 2 (PON2) activity, and thus, the enzymatic antioxidant system is being fulfilled [109].

### 6.6. Protection against Neurodegenerative Diseases

Functional foods and supplements offer great promise to treat neurodegenerative diseases like Alzheimer, Huntington, and Parkinson, and are currently very popular [110]. These types of diseases have specific proteins accumulations, such as prion proteins in the case of Creutzfeldt–Jakob disease and β-amyloid deposition in Alzheimer’s disease that causes oxidative damage to the neurons leading to death. According to Mizrahi et al. [111], punic acid, a derivative of pomegranate seed oil, shows neuroprotective activity by reducing lipid oxidation. Pomegranate phenolics, especially punicalagin, also shows great neuroprotective effects against Alzheimer’s disease [112,113,114]. A study in this regard demonstrates that nanodroplet formulation of pomegranate seed oil can decrease the lipid oxidation, thus stopping the neuronal death in transgenic mouse model of Alzheimer’s disease. Again, long-term supplementation of pomegranate in the diet (for 15 months) has shown improvement in memory and learning and decrease of anxiety in transgenic rats [113]. Ellagic acid, another derivative of pomegranate extract, has been stated for significant reduction of βA1-42-induced neurotoxicity in human cell line [115]. Likewise, quercetin 3-O-glucuronide, another pomegranate extract derivative, is reported to have similar activity in animal models [116]. Pomegranate juice is also reported to be neuroprotective in nature for the neonatal brain. Its supplementation in pregnant mothers’ diet can markedly decrease brain tissue loss and protect neonate against hypoxic-ischemic encephalopathy, as a result of diminished action of caspase-3 [117]. Polyphenolic compounds from pomegranate peel and pulp extracts have also anti-acetylcholinesterase activity, which is beneficial to treat Alzheimer’s, a disease deeply associated with a hyperaction of that enzyme [118]. 

## 7. Pomegranate By-Products as Functional Food Component

Each and every part of this fruit has numerous bioactive compounds with functional as well as medicinal properties. In a recent study, polyphenol-rich plant extracts from pomegranate and red wine were incorporated into the workshop-made cured meat and given to rats (normal and azoxymethane-induced) for 14 and 100 days to evaluate the inhibition of preneoplastic lesions, respectively [119]. Incorporation of plant extracts in cured meat reduces the risk of colorectal cancer linked with processed meats by decreasing the number of mucin-depleted foci per colon. This is due to the suppression of the faecal excretion of nitrosyl iron, considered as precursor of carcinogenesis. Hence, incorporation of pomegranate by-products with high bioactive properties not only improve the quality and shelf life, since inhibit oxidative deterioration (protein and lipid), but also enrich the functional and healthy aspects of foods such as meat, fish, milk, and their products during storage as well [38,120,121,122]. Hereafter, the use of pomegranate by-products as functional ingredients in improving the quality, safety, and functional aspect of muscle foods (meat and fish) is discussed in detail. 

## 8. Pomegranate in Muscle Food Applications

**Figure 3:**
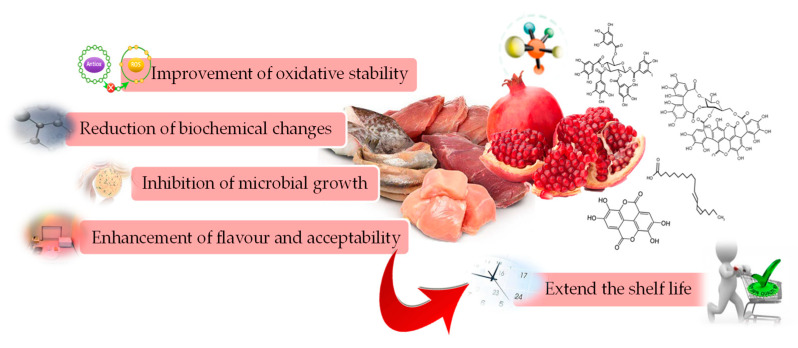
Effect of biomolecules from pomegranate by-products on quality attributes of muscle foods.

Muscle foods (meat and fish), being rich in favourable nutrients, are prone to lipid oxidation, protein decomposition, and microbial contaminations, and spoil rapidly compared to other fresh foods during processing and storage [13,30]. The oxidative changes not only result in accumulation of toxic compounds, but also bring in undesirable changes in the colour, flavour, and texture properties, reducing the acceptability and shelf life of muscle food products [4,12,20,37]. In order to inhibit the undesirable changes and maintain the physicochemical quality and safety of products, bioactive components derived from pomegranate wastes, in the form of powder and or extract, have been used as valuable additives in various muscle food products [30]. Figure 3 also highlights the beneficial effect on quality attributes of muscle foods and associated health benefits of biomolecules derived from pomegranate by-products. 

Many researchers have also indicated the beneficial use of components from pomegranate wastes in preserving colour and aroma, and maintaining the sensory attributes of muscles foods. A summary of various bioactive compounds from pomegranate wastes used in muscle foods and their impact on the quality and safety of prepared products is presented in Table 4. 

### 8.1. Effect on Physicochemical Properties of Muscle Foods

The physicochemical characteristics such as pH, cooking yield, water holding capacity, and chemical composition (moisture, protein, fat, and ash) play an important role in the sensory attributes, quality, and shelf life of muscle foods [4]. Published literature in this regard suggests that pomegranate coproducts such as peel powder, extract, seed power, seed oil, etc., could positively influence the physicochemical properties of muscle foods.

The measurement of pH is one of the important physicochemical characteristics to determine the freshness, which in turn determines the quality and shelf life of meat products. Devatkal et al. [145] reported that the incorporation of pomegranate seed and rind powder decreased the pH of meat patties, mainly due to acidic nature of the powder. Pomegranate rind powder, incorporated at 2%, 4%, and 6% levels, also significantly decreased the pH in buffalo meat (carabeef) nuggets [137]. Likewise, meat samples treated with pomegranate peel extract (PPE) and chitosan–starch (CH–S) composite film incorporated with *Thymus kotschyanus* essential oil had the lowest pH (5.68) values compared to the control (6.65) during storage at 4 °C for a period of 21 days. [146]. While improving the quality of marinated sardine (*Sardinella aurita*) fillets with PPE, Essid et al. [142] reported a considerable dip in pH (from 5.97 to 3.69) of fillets, after 24 h of marination. Again, in a study involving frankfurter, Firuzi et al. [135] reported that incorporation of pomegranate juice concentrates and pomegranate rind powder lowered the pH values compared to control. 

In contrast to these findings, Sharma and Yadav [131] reported nonsignificantly lower pH in both emulsion and cooked chicken patties prepared with pomegranate by-products and extracts. Similarly, no significant difference in pH of cooked chicken patties due to addition of pomegranate rind powder extract was noted by Naveena and his coworkers [147]. However, in all the cases, the pH of muscle foods containing powder and extract from coproducts of pomegranate was in the acceptable range. 

Incorporation of pomegranate peel and seed powder instead of extracts has been reported to improve the emulsion stability and cooking yield in muscle foods [128,131]. The higher cooking yield of muscle food products could be due to the presence of dietary fibres in peel and seed powder and their water and fat binding attributes [4,30]. In a study involving physicochemical composition and functional properties of pomegranate bagasse powder, Viuda-Martos et al. [148] reported that powder derived from pomegranate coproduct (bagasse) exhibited a water holding capacity equal to 4.86 times its own weight. The incorporation of 3% pomegranate rind powder has been reported to improve the water holding capacity in raw beef sausage [59], whereas Abdel Fattah et al. [128] have observed better cooking yield in beef burgers incorporated with pomegranate peel powder. In another study, use of lyophilized pomegranate peel nanoparticles was reported to improve the cooking characteristics such as water holding capacity and cooking yield in beef meatballs [31].

### 8.2. Effect on Colour Attributes of Muscle Foods

Colour is considered as one of the most important meat quality attributes that not only indicates the freshness and quality, but also influences the consumers’ acceptance and rejection of a product [4,24]. According to Hunt and Kropf [149], various factors such as physical characteristics, chemical state of pigments, storage environment and temperature, and presence of nonmeat components play a vital role in the variation of colour attributes of muscle foods. The surface discolouration of muscle foods is associated with metmyoglobin accumulation and is ascribed to the oxidation of ferrous-oxymyoglobin (Fe^2+^) to ferric-metmyoglobin (Fe^3+^), resulting in the production of pro-oxidants that may induce lipid/protein oxidation [150]. Different studies reported the colour stabilizing effect of pomegranate coproducts when they are used in muscle foods. For example, the incorporation of PPE in beef meat steak significantly inhibited discolouration and maintained the most desirable beef colour for longer period compared to the control samples [124]. Concentrated and freeze dried aqueous extract of pomegranate peel also maintained colour intensity (C*) and hue (h*) value of meatballs for 6 months frozen storage [123]. In a similar study, Zhuang et al. [34] reported that the application of aqueous or ethanolic PPE retarded the deterioration of flesh colour of bighead carp (*Aristichthys nobilis*) fillets, attenuating the production of biogenic amines, total volatile basic-nitrogen (TVBN), and the degradation of ATP-related compounds. Likewise, extracts from pomegranate rind powder were reported not only to significantly reduce the surface discolouration by inhibiting the formation of metmyoglobin during storage compared to pomegranate juice, but it was also more efficient in keeping the desirable colour of burgers up to the 90th day of frozen storage [136]. 

Consumers generally reject any muscle food products when there is around 40% metmyoglobin [151]. In a recent study, Fourati et al. [152] reported that minced beef meat treated with 1% PPE had a metmyoglobin value less than 40% until the end of the storage period (21 days). On contrary, meat treated with synthetic antioxidant, butylated hydroxytoluene (BHT), reached its rejection limit (40%) approximately after 14 days of storage. Several researchers have reported the effects of pomegranate coproducts on the colour stabilization, as well as the effects of inhibiting surface discolouring in various muscle food that include frankfurters treated with white and red pomegranate juice concentrates and pomegranate rind powder extract [135], sardine (*S. aurita*) fillets treated with PPE [142], goat meat patties prepared with pomegranate rind powder and pomegranate seed powder [145], Pacific white shrimp (*Litopenaeus vannamei*) treated with PPE [35], beef meatballs incorporated with lyophilized pomegranate peel nanoparticles [31], and raw ground goat meat treated with pomegranate seed powder [126].

### 8.3. Effect on Oxidative Changes of Muscle Foods

The application of natural antioxidants obtained from agrifood wastes on the oxidative changes such as rancid taste and flavour of muscle food products has been reported in several studies [4,153,154,155]. These natural antioxidants apart from preventing the oxidative changes (lipid and protein), extend the shelf life of muscle foods during storage [156,157,158]. Therefore, enriching food products with health-promoting bioactive compounds is good from the consumer point of view [13]. As far as pomegranate is concerned, its coproducts are a good source of phenolic compounds, such as ellagic acid, gallic acids, anthocyanins, and ellagitannins, offering great antioxidant potentiality [40,72]. Interestingly, the antioxidative potential of pomegranate peels are better than the seeds [118]. Even pomegranate peel (5–20 mg tannic acid equivalents/100 g of meat) has more antioxidant potential than those exhibited by BHT in inhibiting lipid oxidation [159]. 

Incorporation of pomegranate by-products in muscle foods has been found to have lower microbial growth, and inhibit lipid and protein oxidation, which otherwise causes quality deterioration such as extensive flavour changes, colour losses, and structural damage to protein, including shorter shelf life and negatively affect the sensory acceptance [120]. For instance, the use of pomegranate fruit by-products like pomegranate rind powder and white and red pomegranate juice concentrate in frankfurters is reported to significantly decrease the thiobarbituric acid reactive substances (TBARS) values by inhibiting lipid oxidation and production of secondary oxidation products. Muscle food containing extracts of pomegranate rind powder presented lower oxidation values than those displayed by samples containing BHT, nitrite, and white and red pomegranate juice concentrate [135]. Beef meatballs treated with 1% PPE had significantly lower malonaldehyde contents and improved shelf life compared to the control. Further, the product had significantly lower peroxide values than control samples, which indicates the potentiality of peel extract to reduce the primary oxidation of meat lipids during storage [11]. Likewise, minced meat of Indian mackerel (*Rastrelliger kanagurta*) treated with PPE at a concentration of 2000 ppm had significantly higher reduction (*p* < 0.05) both in primary and secondary oxidation products [139]. On the other hand, Aliyari et al. [160] noted that TBARS values of cooked beef meat sausages containing different concentrations of PPE were significantly lower than control. PPE was effective in controlling oxidative rancidity and enhancing shelf life of chicken products (chicken chilly and chicken lollipop) by 2–3 weeks during chilled storage [129], and also delayed lipid oxidation in silver carp (*Hypophthalmichthys molitrix*) fish fillet compared to control samples [36]. Furthermore, it was reported that the addition of pomegranate peels to chicken burgers improved the oxidative stability by decreasing peroxide and TBARS values [133]. Various researchers have also indicated that extracts from pomegranate coproducts inhibit primary and secondary lipid oxidation in several meat products, including goat meat patties [145], cooked beef meat sausages [160], chicken breast meat [130], and Tuscan sausages [33]. According to them, the inhibitory effect is due to blocking radical chain reaction in the oxidation process, attributed to their phenolics content [13,161].

Protein oxidation in muscle foods is due to the covalent modification of protein that can be induced directly through ROS or indirectly by secondary products of oxidative stress [20]. ROS can cause oxidation in both amino acid side chains and protein backbones, resulting in protein fragmentation or protein–protein cross-linkages [162]. The concentration of thiol groups decrease due to formation of disulphide bonds, protein aggregates, and cross-links by intermolecular interactions during storage with increasing protein oxidation [19,20]. Oxidative modification affects the structure of muscle proteins and their spatial arrangement by influencing protein net charges and protein cross-linking [163], which negatively influence water holding capacity (WHC) and textural properties of meat products [19,163]. As the WHC and texture are critical quality traits of muscle foods from sensory acceptability as well as economic point view, any change in the structure of muscle proteins and their spatial arrangement may influence these parameters of muscle foods [164]. 

In the case of muscle foods, the protein carbonyl and thiol (sulfhydryl) contents are considered as popular markers to measure the degree of oxidative state of muscle proteins during storage. When muscle food products are subjected to oxidative stress, there will be oxidative degradation of some amino acid side chains, especially lysin, proline, histidine, and arginine residues showing an increased in the protein carbonyl content [165]. It is well known that the major lipid oxidation product is aldehydes and these aldehydes increase protein carbonyls by interacting with muscle proteins [166]. Hence, protein degradation, fragmentation, or aggregation during oxidation process is the major outcome of protein carbonyl formation [154,162]. 

It was found that the extracts of pomegranate peel are efficient on decreasing carbonyl formation and preserving the concentration of sulfhydryl groups in meatballs [123], with loss of total protein solubility and sulfhydryl groups significantly lower in meatballs treated with 1% PEE than control samples during frozen storage. In another study, Fourati et al. [152] reported that minced beef meat treated with PPE had significantly lower carbonyl content at all sampling days than the control samples. Interestingly, beef meat with 1% PPE treatment was found to have the lowest reduction of sulfhydryl group (16.63%), even on day 21 of the storage study. This clearly indicates that pomegranate extract plays a vital role in maintaining the functional properties of protein during storage [152]. In a similar work, inhibitory effect of pomegranate coproducts in decreasing the carbonyl groups was reported by different researchers, notably in raw chicken breasts [125], gutted rainbow trout (*Oncorhynchus mykiss*) [140], and chicken breast meat [130]. 

### 8.4. Effect on Microbiological Quality and Shelf Life of Muscle Foods

Muscle foods, particularly meat and fish, are prone to degradation during storage as a result of microbial activity and/or undesirable chemical reactions [6], hence cannot be stored for long periods. Besides, muscle foods are rich in nutrients, having a high moisture content, and a moderate pH, which also makes them particularly susceptible to microbial contamination [7,8]. The polyphenolic compounds (flavonoids, tannins) from plant components like pomegranate fruit by-products have antibacterial properties. These secondary metabolites exert their inhibitory effect on bacteria by forming complexes with proteins and sulfhydryl groups that make them unavailable for the microorganism [57,59]. A detailed account of antimicrobial properties of pomegranate wastes is mentioned in a previous section. 

Studying the antibacterial potency of PE in meat pâté against *L. monocytogenes* incubated at different temperatures 4, 7, and 120 °C for up to 46 days, Hayrapetyan et al. [167] concluded that PE was effective in inhibiting the microbial growth by 4.1 log CFU/g during 46 days compared to the control, which had reached log 9.2 CFU/g, even on the 18th day of storage. The application of PPE on beef steak surface reduced the bacterial counts for antibiotic resistant strains of *S. aureus*, and hence it could be used for comprehensive meat decontamination and quality-attributes enhancement [124]. Likewise, PE was reported to improve the shelf life of fish patties from ultrafrozen skinless hake fillets (*Merluccius capensis*) in retail display by inhibiting microbial growth and volatile production due to lipid oxidation [121]. Kanatt et al. [129] reported that PPE extract has greater antibacterial potential against *S. aureus* and *Bacillus cereus*, displaying a minimum inhibitory concentration of 0.01%. Furthermore, the authors reported that although higher concentration of extract (0.1%) inhibited Pseudomonas, the extract was not effective against *E. coli* and *Salmonella typhimurium*. Shahamirian et al. [136] reported that meat burgers containing pomegranate juice and rind powder extract had significantly lower aerobic bacterial counts compared to both the control and burgers containing BHT.

Similarly, various researchers have reported that incorporation of pomegranate extract or powder not only exhibited antimicrobial properties but also had improvements in quality in silver carp (*H. molitrix*) fillet [143], beef sausage [138], bighead carp (*A. nobilis*) fillets [34], chicken burgers [133], minced shrimp meat [144], and mackerel fish (*Scomber scombrus*) fillet [141]. These findings clearly indicate that pomegranate peel and seed extract or powder have the potential to be used as natural preservatives in muscle food products. 

### 8.5. Effect on Sensory Acceptability of Muscle Foods 

Sensory attributes such as appearance and colour, texture, juiciness, and flavour are often considered as critical subsets to judge their quality and acceptability, influencing the consumers’ preference and willingness to purchase food products [4,24]. As reported by different workers, components derived from pomegranate by-products exert positive effects on different sensory properties of muscle foods. Incorporation of pomegranate juice or rind powder extract improved the colour (due to presence of red colour anthocyanin) and appearance, as well as preserved other attributes such as flavour, odour, texture, and total acceptance of meat burgers during storage [128,136]. Burgers with PRP received the highest sensory acceptance scores compared to burgers with PJ and BHT, whereas the control sample had relatively very low acceptability, which could be attributed to lipid and protein oxidation. Interestingly, the sourness of PJ reduced the fatty flavour and made the meat burgers pleasant to the panellists [130,136]. Studying the effect of lyophilized pomegranate peel nanoparticles, Morsy et al. [31] reported an improvement in the colour and odour score of beef meatballs, which were acceptable with a high score up to 15 days. Likewise, sardine (*S. aurita*) fillets marinated with PPE had greater colour and appearance scores than control samples [142].

Incorporation of PPP did not have any negative effect on the sensory characteristics of beef sausages [127] and Tuscan sausages [33]. Likewise, the minced meat of Indian mackerel (*R. kanagurta*) treated with pomegranate peel was acceptable up to the eighth day of storage compared to four days for the control samples [139]. Pomegranate-based marinades found to be effective in delaying the spoilage of chicken breast fillets and improving the sensory characteristics [132]. Again, silver carp (*H. molitrix*) fillets had better acceptability (up to 12 days) compared to control samples (9 days) when they were treated with 5% and 10% PPE. In general, the incorporation of pomegranate by-products into muscle foods as natural preservatives improved colour, flavour (odour score), and overall acceptability, and extended their shelf life during storage. 

## 9. Conclusions and Future Perspectives

The quantity and quality of bioactive components present in pomegranate wastes (phenolic and polyphenolic compounds, dietary fibre, complex polysaccharides, minerals, vitamins, etc.) depends on many factors such as variation among varieties, climatic conditions, cultivars, developmental stages, and extraction processes. In addition, several methods/techniques such as ultrasound, microwave, supercritical fluid, pulse electric field, high pressure, ohmic, UV, and infrared heating are currently being applied for the extraction of bioactive components from pomegranate wastes [168]. In this regard, the incorporation of pomegranate by-products with high bioactive properties into foods such as meat, fish, and milk is a good strategy to obtain functional foods and improve their quality and shelf life during storage.

## Figures and Tables

**Table 1:** Proximate composition, major micronutrients, vitamins, and polyphenolic content of pomegranate peel and seed powder.

Parameters	Pomegranate Peel	Pomegranate Seed Powder
Moisture (%)	13.7	5.8
Protein (%)	3.1	13.7
Fat (%)	1.8	29.6
Ash (%)	3.3	1.5
Fibre (%)	11.2	39.4
Carbohydrates (%)	80.5	13.5
Calcium (mg/100 g)	338.50	229.20
Potassium (mg/100 g)	146.40	434.40
Sodium (mg/100 g)	66.43	33.03
Phosphorus (mg/100 g)	117.90	481.10
Iron (mg/100 g)	5.93	10.88
Vitamin C (mg/100 g)	12.90	3.02
Vitamin E (mg/100 g)	3.99	1.35
Total polyphenol (mg/g GAE)	53.65 (WE)	7.94 (WE)
	85.60 (ME)	11.84 (ME)
Total flavonoids (mg/g TE)	21.03 (WE)	3.30 (WE)
	51.52 (ME)	6.79 (ME)
Total anthocyanins (mg/g CGE)	51.02 (WE)	19.62 (WE)
	102.02 (ME)	40.84 (ME)
Hydrolysable tannins (mg/g TAE)	62.71 (WE)	32.86 (WE)
	139.63 (ME)	29.57 (ME)

GAE: gallic acid equivalents, TAE: tannic acid equivalents, CGE: cyanidin-3-glucoside equivalents, WE: water extract, ME: methanol extract. Source: [29,45,46].

**Table 2:** Pomegranate plant parts and their chemical constituents.

Pomegranate Plant Parts	Chemical Constituents
Pomegranate juice from the succulent testa	Glucose, ascorbate, ellagic acid, anthocyanins, caffeic acid, catechin, quercetin, amino acids and minerals.
Seed oil	Punicic acid, ellagic acid, sterols, phytoestrogens
Peel (pericarp)	Phenolic punicalagins, gallic acid, catechin, flavones, etc.; Flavonoids (catechin, flavan-3-ol, epicatechin, quercetin, kaempferol, rutin, kaempferol 3-O-glycoside, kaempferol 3-O-rhamnoglycoside, naringin epigallocatechin 3-gallate, luteolin, and luteolin 7-O-glycoside); Ellagitannins (punicalagin, punicalin, corilagin, gallagyldilacton, tellimagrandin, casuarinin, pedunculagin, granatin A, and granatin B); Pelletierine alkaloids (pelletierine); caffeic acid; p-coumaric acid; chlorogenic acid; quinic acid; polyphenols (saponins, ellagic tannins, ellagic acid, and gallic acid); anthocyanidins; additionally, triterpenoids, steroids, glycosides, carbohydrate, vitamin C, ascorbic acid, and tannins
Leaves	Tannins, flavone glycosides, luteolin, apigenin
Flowers	Gallic acid, urosolic acid, triterpenoid compounds, including maslinic and asiatic acid
Bark and roots	Punicalin, punicalagin, piperidine alkaloids, ellagitannins

Source: [29,45,46].

**Table 4:** A summary of the various bioactive compounds from pomegranate wastes used in muscle foods and their impact on the quality and safety of prepared products.

Muscle Foods	Pomegranate Products and Level of Use	Parameters Studied and Storage Conditions	Key Findings	Ref.
Meat and Meat Products				
Turkish meatballs	Lyophilized water extract of pomegranate in meatballs—0.5 and 1%	Lipid and protein oxidation of meatballs stored at refrigerated conditions (4 ± 1 °C) for 8 days	Lowered lipid (TBARS value, peroxide formation) and protein (formation of protein carbonyls) oxidation Improved sensory scores (colour and rancid odour) and prolonged the refrigerated storage of meatballs up to 8 days compared to control	[11]
Beef meatballs	Lyophilized pomegranate peel nanoparticles—1 and 1.5%	Antioxidant and antimicrobial effects of meat balls during storage at 4 ± 1 °C up to 15 days	Effectively retarded the lipid oxidation Improved the cooking (WHC and cooking yield) and sensory characteristics of meatballs during storage up to 15 days	[31]
Tuscan sausages	Pomegranate peel extract—0.025, 0.5 and 0.1%	Lipid stability of fresh Tuscan sausages stored at a mean temperature of 5 ± 1 °C for 30 days	Delayed lipid oxidation demonstrating stability of the manufactured Tuscan sausages Showed an adequate global acceptability level in the sensory analysis	[33]
Beef meatballs	Concentrated and freeze-dried aqueous extract of pomegranate peel (PP)—0.5% and 1.0%	Antioxidant effect of PP in beef meatballs during 6 months of frozen storage at −18 ± 1 °C	PP lowered the carbonyl formation, loss of total protein solubility and sulfhydryl groups in beef meatballs than control samples Maintained the colour intensity and hue value Effective as natural antioxidant in preventing rancid odour formation	[123]
Beef meat steak	Pomegranate peel extract —250 µg/mL	Decontamination of meat steak surfaces and sensory attributes after 7 days of storage at 4 °C	Significantly lowered the bacterial counts of antibiotic resistant strains of *Staphylococcus aureus* on meat steak surface Maintained the desirable sensory attributes with minimum odor alterations compared to others. Prevented surface discoloration in meat steaks by maintaining the beef color	[124]
Raw chicken breasts	Pomegranate fruit juice—0.02%	Physicochemical and sensory attributes of samples packed and stored at 4 °C for 28 days	Reduced the protein oxidation Inhibited microbial growth Increased sensory acceptability for up to 12 days	[125]
Raw ground goat meat	Pomegranate seed powder—2%	Color and oxidative stability of raw ground goat meat stored aerobically at 4 °C ± 1 °C for 6 days	Lowered the TBARS values and decreased the lipid oxidation by about 80% in treated sample compared with control Significantly improved the redness s and decreased the brightness values in treated sample	[126]
Beef sausage	Pomegranate peel powder —1%, 2%, and 3%	Keeping quality and sensory attributes of sausages during storage at (4 ± 2 °C) for 12 days	Reduced the production of TBA and TVN of samples during storage compared to control Improved cooking quality viz., cooking loss, cooking yield, and no negative effects on the sensory characteristics of the product	[127]
Beef burger	Pomegranate peel powder —1%, 2%, and 3%	Keeping quality and safety characteristics of burger during a storage period at 4 ± 1 °C for 12 days.	Improved the cooking characteristics e.g., cooking loss, cooking yield, and change in diameter Improved the storage stability by significantly reducing TBARS and TVN production Had positive effects on the sensory characteristics of the product	[128]
Chicken products (chicken chilly and chicken lollipop)	Pomegranate peel extract —0.01%, 0.05%, and 0.1%	Antioxidant and antimicrobial potential of products during chilled storage conditions	Showed good antimicrobial activity against *S. aureus* and *Bacillus cereus* having minimum inhibitory concentration of 0.01% Inhibited *Pseudomonas* sp. at a higher concentration of 0.1% but was ineffective against *Escherichia coli* and *Salmonella typhimurium* Effective in controlling oxidative rancidity and enhancing shelf life by 2–3 weeks during chilled storage	[129]
Chicken breast meat	Pomegranate juice—2%	Chemical, sensorial, and microbiological analyses of chicken breast meat stored at 4 °C for 20 days	Significantly decreased total viable count, lactic acid bacteria, Enterobacteriaceae, psychrotrophic bacteria, yeasts, and molds compared to control Significantly lowered the peroxide value, TBARS, and protein oxidation in treated sample compared to control	[130]
Chicken meat patties	Pomegranate peel powder (2 g), pomegranate aril bagasse powder (PABP, 4 g), pomegranate peel powder aqueous extract (6 g) and pomegranate aril bagasse powder aqueous extract (9 g)	Quality characteristics and shelf life of chicken patties during storage	Increased the ash, crude fibre, and hardness values, and significantly decreased the moisture content and lightness values compared to control patties PABP improved the emulsion stability and cooking yield of treated patties compared to control Both the powder and extract forms provided better protection against oxidative rancidity and microbial proliferation compared to control and BHT-treated patties during storage	[131]
Chicken breast fillets	Pomegranate juice (70%) based marinades	Microbiological and sensory analyses of samples marinated at 4 °C for 3 h and aerobically stored at 4 and 10 °C for 9 days	Inhibited Pseudomonads and *Brochothrix thermosphacta* and reduced production of volatile compounds and organic acids that can cause off-odors Significantly extended the shelf life of fillets up to 5 and 6 days at both storage temperatures Effective in delaying the spoilage of chicken meat and improving the sensory characteristics.	[132]
Chicken burgers	Pomegranate peels—10%, 20%, and 30% (*w*/*w*)	Microbial load and shelf life of chicken burgers during cold storage (4 ± 1 °C) for 2–10 days	Decreased the production peroxide and TBA values and retarded the growth of total molds and yeasts, psychrophilic and spore-forming bacteria Improved the appearance, odor, texture, and taste of the chicken burgers samples	[133]
Fat rich mutton products (Tabaq-Maz)	Pomegranate rind extract (PRE)—0.5%, 1.0%, and 1.5%	Microbiological profile and sensory evaluation of mutton products dipped in PRE for 30 s and packaged in low density polyethylene pouches and stored at 4 ± 1 °C for 21 days	PRE (1.0% and 1.5%) improved the lipid oxidative stability and microbial quality of the products. The products retained good sensory scores up to 14th day at refrigerated storage (4 ± 1 °C) conditions PRE (1.0% and 1.5%) could be used as a novel natural preservative in fat rich meat products like Tabaq-Maz	[134]
Frankfurter	White and red pomegranate juice concentrates and pomegranate rind powder extract (equivalent to 10 mg gallic acid at 100 g sample)	Oxidation indices, pH, microbial quality and color of frankfurter samples during storage at 4 °C for 60 days	Delayed the oxidation process, thereby reducing the peroxide value and TBARS values compared to control Improved the color of frankfurter samples Pomegranate rind powder was more effective than pomegranate juice in reducing oxidation in cooked chicken patties	[135]
Frozen meat burgers	Pomegranate rind powder extract (PRPE) and pomegranate juice (PJ)—100 ppm	Oxidative stability, sensorial and microbiological characteristics of burgers stored at −18 °C for 90 days	PRPE showed higher antioxidant capacity and better protective effect on lipids in burgers than PJ. Both PJ and PRPE lowered the aerobic bacterial growth in burgers than the control PRPE maintained highest values (scores) in terms of sensory attributes and acceptability of the products compared to others	[136]
Buffalo meat (carabeef) nuggets	Pomegranate rind powder (PRP)—2, 4 and 6%	Physicochemical, sensory attributes, and antioxidant capacity of nuggets packed under aerobic and vacuum conditions and stored at refrigerator temperature for up to 14 days	Significantly decreased the pH, emulsion stability, cooking yield, crude protein, ether extract, ash content and moisture content with increase in PRP level Significantly increased the crude fiber and antioxidant capacity Improved the physicochemical characteristics, sensory properties and lipid stability of treated nuggets during storage Nuggets with 4% PRP considered as the best in terms of overall acceptability as per the sensory score.	[137]
Beef sausage	Pomegranate peel powder (PPP)—2.5% and 5%	Physicochemical and microbiological effects of PPP on beef sausage stored at –18 ± 2 °C for 8 weeks	PPP had a substantial effect on pH, TVN and TBA over the storage period compared to the control group Significantly reduced the TBC and Enterobacteriaceae counts in treated group	[138]
Fish and shellfish products				
Bighead carp (*Aristichthys nobilis*) fillets	Aqueous pomegranate peel extract (APPE) and ethanolic pomegranate peel extract (EPPE) (equivalent 0.5 mg GAE/mL)	Changes in microbiota and quality of fillets aerobic packaged in high-density polyethylene bags and stored at 4 °C for 8 days	EPPE performed better in color attributes and biogenic amines, but APPE was more effective in retarding the increase of TVB-N and K-value APPE had a better inhibitory effect on Aeromonas, but EPPE was better at inhibiting Pseudomonas EPPE preferred in terms of colour stability and extending shelf life for about 2 days	[34]
Silver carp (*Hypophthalmichthys molitrix*) fillet	Pomegranate peel extract (PPE)—5% or 10%	Quality and shelf life of PPE coated silver carp fillet during refrigerated storage	Delayed lipid oxidation in fillets compared to control samples. Reduced TVC and psychrotrophic count Treated samples had better acceptability (up to 12 days) compared to control (9 days)	[36]
Ultra-frozen skinless hake fillets (*Merluccius capensis*)	Pomegranate extract—200 ppm	Physicochemical and microbiological characteristics of samples aerobically packed and stored at 4 °C until 11 days.	Delayed the lipid oxidation, production of volatile compounds, and microbiological spoilage in fish fillets Extended the shelf life of fish under retail display conditions	[121]
Minced meat of Indian mackerel (*Rastrelliger kanagurta*)	Pomegranate peel extract (PPE)—1000, 1500, and 2000 ppm	Effect on lipid oxidation in cooked meat stored at 4 ± 2 °C for 8 days	PPE (2000 ppm) showed significantly higher reduction (*p* < 0.05) in primary and secondary oxidation products Extended the shelf life of minced meat from 4 days to 8 days of storage. PPE effective as a natural antioxidant for controlling the oxidative rancidity in fish and fishery products	[139]
Gutted rainbow trout (*Oncorhynchus mykiss*)	Dipping fish in methanolic pomegranate peel extract (MPPE)—1, 2, or 4% (*w*/*v*)	Microbiological, chemical, sensory, and textural characteristics of samples stored at 18 °C for 6 months	MPPE (4%) inhibited the protein oxidation The highest score for general acceptability achieved with 1% MPPE Greater hardness and chewiness observed with 4% MPPE Dipping fish in MPPE an effective method to extend the shelf life and the overall quality of the product	[140]
Mackerel fish (*Scomber scombrus*) fillet	Pomegranate peel extract (PPE)—2.5%, 5%, 7.5%, or 10% in 3% alginate solution	Antibacterial effect of PPE coating in fillets against food borne pathogens stored at 4 ± 1 °C for 13 days	Significantly (*p* < 0.05) decreased *Listeria monocytogenes* in fillets Significantly lowered the total aerobic mesophilic bacteria and Enterobacteriaceae number compared to the control sample	[141]
Sardine (*Sardinella aurita*) fillets	Pomegranate peel extracts (PPE)—5%	Biochemical, microbiological and sensorial quality of PPE marinated fillets stored for 90 days	PPE marinated fillets showed better oxidative stability and higher content of PUFA and significantly decreased TVB-N and TMA during storage compared to control Control samples had higher values of FFA and histamine Samples marinated with PPE had greater color and appearance scores than the control samples	[142]
Silver carp (*H. molitrix*) fillet	Encapsulated and unencapsulated pomegranate peel extract—0.5% and 1%	Effects of extract on the antimicrobial and antioxidant activities of fillets stored at 4 °C	Reduced chemical deterioration and lipid oxidation as reflected with lower TVBN and TBA values. Inhibited TVC of the fillets compared with control	[143]
Pacific white shrimp (*Litopenaeus vannamei*)	Pomegranate peel extract (PPE)—1.5%	Effect of PPE on the melanosis and quality of shrimp during 10 days of iced storage	Significantly inhibited the melanosis in shrimp compared to the control Delayed the change of color and significantly prevented the decrease in sensory scores of shrimps than control PPE could be an alternative to synthetic antimelanosic agents to inhibit postmortem melanosis and improve the quality of shrimp during iced storage	[35]
Minced shrimp meat	Pomegranate peel ethanolic extracts (PoPetx)—0.05, 0.1, or 0.2 g/10g meat	In situ efficacy of hydroalcoholic PPE in controlling microbial growth and lipid oxidation of shrimp meat stored at 4 °C for 28 days	Showed microbial inhibitory action against TPC and *Staphylococcus* spp. Preserved the chemical quality of shrimp during storage	[144]

BHT: butylated hydroxytoluene; FFA: free fatty acid; PUFA: polyunsaturated fatty acid; TBA: thiobarbituric acid; TBARS: thiobarbituric acid reactive substances; TBC: total bacterial count; TMA: trimethylamine; TVC: total viable count; TVN: total volatile nitrogen; WHC: water holding capacity.
